# Differences in quality of life among older adults in Brazil according to smoking status and nicotine dependence

**DOI:** 10.1186/s12955-018-1072-y

**Published:** 2019-01-03

**Authors:** Dayane Aparecida Viana, Flavia Cristina Drumond Andrade, Luiz Claudio Martins, Leiner Resende Rodrigues, Darlene Mara dos Santos Tavares

**Affiliations:** 1 Gerontology program, Universidade Estadual de Campinas, Rua Tessália Vieira de Camargo, Campinas, SP 126 Brazil; 2 School of Social Work, University of Illinois at Urbana-Champaign, 1010 W Nevada St, Urbana, IL 61801 USA; 3 Nursing, Education and Community Health Department, Universidade Federal do Triângulo Mineiro – Uberaba, Av. Getúlio Guaritá, Uberaba, MG 107 Brazil

**Keywords:** Quality of life, Smoking, Aging, Brazil

## Abstract

**Background:**

Research on quality of life QOL is limited in Brazil and few studies have examined the association between smoking status and quality of life. This study addresses this gap and also examines the association between smoking, nicotine dependence, and duration of smoking cessation on (QOL) among older adults in an urban area in Brazil.

**Methods:**

Data are from a household survey conducted in urban areas of Uberaba, Brazil, in 2012 (*n* = 980). Multivariable linear regressions were used to evaluate the association between smoking, nicotine dependence based on Fageström test, and smoking cessation on the World Health Organization Quality of Life WHOQOL-BREF and Quality of Life Assessment for Older Adults WHOQOL-OLD.

**Results:**

The mean age of older adults in the study was 74.0 (SD = 6.9 years) and 64% of participants were women. The majority, 55% had never smoked, 12.4% were current smokers, and 32.7% were past-smokers. Current smokers had lower scores for social participation (β = − 2.6) and intimacy (β = − 3.8) than never smokers. Smokers with high or very high dependence reported higher levels of fear and concern about death and pain before death than those with low or very low dependence (β = − 10.6). However, smokers with medium levels of nicotine dependence had higher scores on social relationship. Longer cessation time was positively associated with higher scores for psychological health.

**Conclusions:**

Except for the positive association between medium levels of nicotine dependence and better social relationships, smoking and higher levels of nicotine dependence were associated with worse QOL among older adults in Brazil. Nonetheless, smoking cessation had positive effects in QOL. Campaigns targeting older adults should point to the negative impact of tobacco use on QOL and the benefits of smoking cessation.

## Background

The Brazilian population aged 60 and over is expected to grow almost four-fold in the next 50 years from 19.6 million in 2010 to 73.5 million by 2060 [1]. This change is associated with changes in quality of life (QOL) including, but not limited to, a greater prevalence of chronic conditions and disability. Unhealthy lifestyle choices, such as tobacco use, may exacerbate these health changes and impact QOL [2]. Data from the survey VIGITEL-Brazil show that 9.8% of individuals aged 55 to 64 residing in Brazilian state capitals and the Federal District are smokers, and 7.7% of those aged 65 years and over also smoke [3]. Prevalence rates of smoking are higher among older men than women in Brazil [4]. While the prevalence of smoking is similar among older men of different educational levels in Brazil, older women with higher education have higher prevalence rates, which reflect past perceptions of smoking as a symbol of freedom [5]. The overall prevalence of smoking in Brazil has been declining since the end of 1980s [6], and Brazil has been very successful in reducing smoking related deaths [7]. Today, smoking cessation is associated with fewer hospitalizations, lower incidence of chronic conditions, improvements in functional status, increased survival, and better QOL for adults over 60 [8–10]. Nonetheless, smoking is still responsible for about 147,000 deaths annually in Brazil [11].

Given the increase in life expectancy in Brazil [12], more attention has been devoted to older adults’ QOL, which includes personal beliefs, relationships and interactions with one’s environment as well as physical and mental health [13]. Since research on QOL is limited in Brazil, few studies have examined the association between smoking status and QOL [14–16]. Previous studies in Brazil have not included measures of nicotine dependence, even though nicotine addiction varies among smokers and differential exposure to smoking can cause serious dysfunction in the body, leading to functional disability and a lower QOL [17]. We address this gap in the literature by examining the associations between smoking, nicotine dependence, and duration of smoking cessation on QOL among older adults in an urban area in Brazil. In addition, this study also uses the Quality of Life Assessment for Older Adults WHOQOL-OLD, which is a measure of QOL developed for older adults to examine the QOL among older adults, in addition to the World Health Organization Quality of Life WHOQOL-BREF, which has been previously used [18].

## Methods

### Data

Data are from a household survey conducted in urban areas of Uberaba, Brazil, in 2012. The study began in 2008 with a sample size calculation of 3034 older adults. Between June and December of 2012, trained interviewers visited the homes of 2149 older adults. Of these, 980 individuals met the inclusion criteria of this study: age of 60 years or older, resident in the urban area of the municipality, and absence of cognitive impairment. Exclusions and/or losses of participants were related to death (*n* = 266), cognitive impairment (*n* = 160), and other reasons such as refusals, problems locating, and hospitalizations (*n* = 743). Details about sample selection have been published elsewhere [19, 20].

After obtaining informed consent, researchers administered the Mini-Mental State Questionnaire (MMSE) to participants [21]. The MMSE evaluates the cognition health and it has been translated and validated in Brazil [22]. The test provides a score of 0–30. Given the low levels of education among older adults in Brazil, specific cut-off points are used based on the schooling level of the older adults: 13 for illiterate people, 18 for those with 1–11 years of schooling, and 26 for those with more than 11 years of schooling [22]. Average MMSE was 23.5 (SD 3.8). Participants who evidenced cognitive impairment based on Brazilian-specific cutoff were excluded from the study.

### Variables

The QOL assessed by using the WHOQOL-BREF and WHOQOL-OLD, which have been translated and validated in Brazil [23, 24]. The abbreviated WHOQOL-BREF provides scores for four domains related to QOL: physical health, psychological, social relationships and environment [25]. The WHOQOL-BREF consists of 26 items rated on a 5-point Likert scale. The response options range from 1 (very dissatisfied/very poor) to 5 (very satisfied/very good) [23]. The WHOQOL-OLD recognizes specific areas of quality of life that could be more important for older people [18]. The WHOQOL-OLD consists of 24 items rated on a 5-point Likert scale. The questionnaire addresses six facets: sensory abilities; autonomy; death and dying; past, present, and future activities; social participation; and intimacy. Each facet contains four items. After reverse coding items from the sensory abilities facet and death and dying, the transformed scores are obtained. In both questionnaires the scores are transformed and vary from 0 to 100, with higher scores representing higher levels of QOL [24].

Smoking was classified using the Guidelines for Smoking Cessation [26]: current smokers (those who reported having smoked at least one cigarette per day for the last 6 months), past-smokers (those who have smoked in the past, but are not current smokers), and never-smokers (those who have never smoked). The Nicotine Dependence Test of Fageström, translated and validated in Brazil [27], was used to evaluate the degree of nicotinic dependence among older adults who smoked. This test assesses the intensity of physical addiction to nicotine using six questions that assess the quantity of cigarette consumed, the compulsion to use cigarettes, and dependence [28]. Scores range from 0 to 10 with higher scores indicating higher physical dependence to nicotine. Individuals were classified into groups: very low dependence (0 to 2), low dependence (3 to 4), medium dependence (5), high dependence (6 to 7), and very high dependence (8 to 10). Because of the distribution of groups, those with very low and low dependence were combined, as were those with high and very high dependence. In addition to smoking status and nicotine dependence, we collected three other variables for the analysis: number of cigarettes per day, smoking exposure (in years), and smoking cessation (in years).

Sociodemographic variables included gender (male or female), age group (60 to 69 years, 70 to 79 years, and 80 years or more), education (illiterate, 1–4 years of schooling, and 5 or more years of schooling), and number of health conditions (0–4 and 5 or more health conditions).

### Statistical analysis

Table 1 provides descriptive statistics, such as frequencies and percentages. Tables 2 and 3 provide the ANOVA test used to compare the scores of WHOQOL-BREF AND WHOQOL-OLD across both categories of smokers and of nicotine dependence. We also present the results for the Bonferroni method that allows for pairwise comparisons. Multivariable linear regression models adjusted for age, sex, education, and health conditions were used to examine the association between smoking indicators and QOL. Table 4 presents the results of the multivariate regressions that examines the association between QOL and smoking status. In Table 5, the sample is restricted to those who are currently smokers who provided information on nicotine dependence. The results in Table 5 focus on the association between nicotine dependence and QOL. Finally, Table 6 focuses on former smokers and examines whether duration of cessation is associated with QOL. Data were analyzed using STATA/SE 14.0.

**Table 1:** Socio-demographic and smoking characteristics of the older adults in Uberaba, Brazil

Variables	Smoker		Past smoker		Never smoker		Total	
	n	(%)	n	(%)	n	(%)	n	(%)
Sex								
Male	53	15.2	188	53.9	108	30.9	349	35.6
Female	69	10.9	132	20.9	430	68.1	631	64.4
Age group								
60 to 69 years	56	19.8	77	27.2	150	53.0	283	28.9
70 to 79 years	47	9.6	170	34.7	273	55.7	490	50.0
80 years or more	19	9.2	73	35.3	115	55.6	207	21.1
Education (years of schooling)								
Illiterate	32	15.1	68	32.1	112	52.8	212	21.7
1 to 4 years	63	11.5	193	35.2	293	53.4	549	56.1
5 or more	27	12.4	58	26.6	133	61.0	218	22.3
Health conditions								
0–4	56	14.1	137	34.5	204	51.4	397	40.6
5 or more	66	11.3	183	31.4	333	57.2	582	59.5
Nicotine dependence								
Very low/Low	69	56.6	–	–	–	–	–	–
Medium	24	19.7	–	–	–	–	–	–
High/Very high	29	23.8	–	–	–	–	–	–
Cigarette consumption								
Cigarettes / day	122	12.7, 10.7	320	18.5, 15.4	–	–	–	–
Time of exposure to tobacco (in years)	122	49.5, 15.0	320	27.8, 16.8	–	–	–	–
Time since stopped smoking (in years)	–	–	320	24.0 14.71	–	–	–	–

**Table 2:** Descriptive statistics (mean ± SD) of WHOQOL-BREF and WHOQOL-OLD, by smoking categories, Uberaba, Brazil

QOL	Smoking Categories						
	Smokers		Ex-smokers		Never smoker		p
	Mean	SD	Mean	SD	Mean	SD	
WHOQOL-BREF							
Physical	61.27	16.39	61.90	17.39	61.53	17.10	0.925
Psychological	64.82	14.30	67.52	15.54	65.73	15.99	0.156
Social relationship	70.49	15.34	72.44	14.01	71.17	14.22	0.318
Environmental	59.81	12.66	61.35	13.89	60.60	14.27	0.543
WHOQOL-OLD							
Sensory abilities	67.41	23.69	69.86	24.04	68.70	23.45	0.593
Autonomy	62.80	15.07	65.56	15.76	64.33	15.73	0.233
Past, present and future activities	66.90	14.04	68.20	15.93	67.38	15.40	0.661
Social participation	62.70	14.83	65.64	16.86	65.05	16.27	0.232
Death and Dying	73.03	22.73	73.51	25.03	70.44	26.91	0.204
Intimacy	65.67	21.96	69.70	19.78	68.06	20.35	0.164

**Table 3:** Coefficients of multivariable linear regression examining the association between smoking categories and quality of life indicators. Uberaba, Brazil

	WHOQOL-BREF				WHOQOL-OLD					
Variables	Physical	Psychological	Social relationship	Environmental	Sensory abilities	Autonomy	Past, present and future activities	Social participation	Death and Dying	Intimacy
Smoking categories										
Never smoker (ref)										
Past smoker	−0.1	0.1	−0.0	− 0.4	1.9	0.6	0.1	− 0.5	1.9	−0.6
Current smoker	−1.1	− 1.9	− 1.0	−1.1	−1.7	−2.1	− 0.8	−2.6*	1.8	−3.8*
Sex										
Female (ref)										
Male	0.4	4.0***	2.6**	3.0***	−1.8	1.5	1.8	2.3*	2.0	5.9***
Age groups										
60–69 years	2.0	− 0.6	−2.4*	−2.4*	5.4**	2.1	0.5	−0.0	−1.8	− 1.2
70–79 years	2.8**	0.6	−0.0	−0.2	3.7*	2.3*	2.1*	3.6***	−1.4	− 1.0
80 years or more (ref)										
Education										
Illiterate	−6.2***	−4.5***	−0.8	−7.5***	− 6.4***	−4.2***	−2.8*	−4.2***	2.4	−4.4**
1 to 4 years	−4.7***	−3.4***	0.1	−3.9***	−4.0**	− 1.7	− 2.3*	− 2.1	1.2	−4.3***
5 or more (ref)										
Health conditions										
0–4 (ref)										
5 or more diseases	−12.3***	−4.8***	−2.1**	−3.7***	− 7.7***	−3.5***	− 1.9*	− 4.2***	− 6.0***	− 1.9
Constant	70.9***	70.5***	72.8***	66.8***	73.9***	66.4***	68.9***	67.4***	73.7***	72.3***
R^2^	0.1545	0.0618	0.0220	0.0718	0.0435	0.0328	0.0176	0.0460	0.0199	0.0360

Note: ref- reference categories

*P < 0.10; **P < 0.05; ***P < 0.01

**Table 4:** Descriptive statistics (mean **±** SD) of WHOQOL-BREF and WHOQOL-OLD, by degree of nicotine dependence, Uberaba, Brazil

QOL	Very Low/Low		Medium		High/Very High		p
	Mean	SD	Mean	SD	Mean	SD	
WHOQOL-BREF							
Physical	61.23	16.70	62.20	15.33	60.59	17.03	0.939
Psychological	64.61	13.40	67.01	13.79	63.51	16.91	0.666
Social relationship	69.81	15.13	75.00	15.73	68.39	15.33	0.254
Environmental	59.60	13.01	60.81	8.74	59.48	14.79	0.912
WHOQOL-OLD							
Sensory abilities	66.94	23.36	70.05	26.13	66.38	23.05	0.829
Autonomy	62.32	14.10	61.72	15.56	64.87	17.15	0.693
Past, present and future activities	66.58	13.47	65.63	16.38	68.75	13.67	0.695
Social participation	61.87	14.97	64.32	16.84	63.36	13.02	0.757
Death and Dying	75.27	20.93	72.66	26.95	68.10	22.74	0.360
Intimacy	64.04	21.56	65.36	21.33	69.83	23.63	0.494

**Table 5:** Coefficients of multivariable linear regression examining the association between nicotine dependence, smoking duration and quality of life among current smokers. Uberaba, Brazil

Variables	WHOQOL-BREF				WHOQOL-OLD					
	Physical	Psychological	Social relationship	Environmental	Sensory abilities	Autonomy	Past, present and future activities	Social participation	Death and Dying	Intimacy
Nicotine dependence										
Very low or low (ref)										
Medium	3.0	3.8	6.5*	2.9	5.2	−0.6	0.1	2.9	−4.0	1.9
High or very high	−0.0	−1.2	0.1	1.0	−0.7	3.4	2.8	1.4	−10.6**	4.9
Exposure to smoking in years	−0.2**	− 0.1	− 0.0	−0.1	− 0.1	− 0.1	− 0.1	− 0.1	− 0.0	− 0.3*
Sex										
Female (ref)										
Male	−0.1	2.7	− 0.8	− 0.6	−1.6	1.5	− 1.8	2.7	8.8*	7.3
Age groups										
60–69 years	−6.4	−10.0**	−6.5	−8.5**	−7.1	0.9	−5.6	−2.7	2.3	−10.9
70–79 years	−3.1	−6.3	1.8	−0.1	−3.8	6.3	−1.7	4.1	2.4	−1.5
80 years or more (ref)										
Education										
Illiterate	−2.9	− 2.3	−3.4	− 7.0**	− 8.0	−1.8	−1.1	− 2.6	− 2.8	−0.1
1–4 years of schooling	0.4	5.4*	5.3*	−0.4	− 7.8	1.3	−1.1	− 1.5	−9.2**	2.3
Health conditions										
0–4 (ref)										
5 or more diseases	−13.7***	−7.3***	−4.3	−0.7	−9.2**	−1.5	0.6	−5.5**	−5.6	0.1
Constant	84.9***	77.1***	73.8***	71.0***	87.1***	64.8***	74.2***	71.1***	79.3***	79.0***
R^2^	0.2474	0.1647	0.1399	0.1373	0.0732	0.0543	0.0340	0.1004	0.1176	0.0701

Note: ref. – reference categories. *P < 0.10; **P < 0.05; ***P < 0.01

**Table 6:** Coefficients of multivariable linear regression examining the association between smoking cessation time a QOL indicators. Uberaba, Brazil

Variables	WHOQOL-BREF				WHOQOL-OLD					
	Physical	Psychological	Social relationship	Environmental	Sensory abilities	Autonomy	Past, present and future activities	Social participation	Death and Dying	Intimacy
Cessation in years	0.1	0.2***	0.1	0.1	0.1	0.0	0.1	0.0	0.1	0.1
Sex										
Female (ref)										
Male	0.2	4.5**	3.5**	3.7**	−4.1	0.2	0.8	2.4	2.1	5.8**
Age groups										
60–69 years	0.9	−1.1	−2.8	−2.1	6.3	1.1	1.5	−0.6	−2.9	0.4
70–79 years	3.0	2.2	−1.1	−0.8	2.0	2.8	2.1	3.2	−1.0	0.4
80 years or more (ref)										
Education										
Illiterate	−5.6*	−3.6	0.2	−7.3**	−5.0	−6.4**	−2.3	−4.6	5.9	−1.4
1–4 years	−6.4**	−5.9***	−0.2	−6.1**	−1.3	−6.0**	−4.2	−3.6	4.6	−3.9
Health conditions										
0–4 (ref)										
5 or more diseases	−11.7***	−4.0**	−2.0	−3.0*	−7.4***	− 3.6**	−0.8	− 4.1**	−4.7	− 1.5
Constant	70.4***	66.8***	71.6***	65.2***	73.6***	69.8***	67.5***	67.5***	70.1***	66.8***
R^2^										

Note: ref. – reference categories. *P < 0.10; **P < 0.05; ***P < 0.01

## Results

The mean age of older adults in the study was 74.0 (SD = 6.9 years) and most participants were women (64%). The majority, 538 (54.9%), had never smoked, 122 (12.4%) were current smokers, and 320 (32.7%) were past-smokers (Table 1). Men were more than twice as likely to have smoked as women. Among those who currently smokers, the consumption of cigarettes is 12.7 cigarettes/day on average. About 27% of current smokers have a high or very high degree of nicotine dependence. On average past smokers had been nicotine free for 24 years and were exposed for 27.8 years (Table 1).

Results presented in Table 2 show the average scores and standard deviations for WHOQOL-BREF and WHOQOL-OLD by smoking categories. One-way ANOVA was used to assess the differences across these groups. Results from ANOVA show no statistically significant differences on QOL scores by smoking status. In addition, Bonferroni multiple-comparison tests did not indicate statistically significant differences when comparing each pair of smoking categories.

The following analyses, presented on Table 3 focus on current smokers who provide information on smoking dependence. ANOVA results presented indicate no statistically significant differences on scores across levels of smoking dependence. Bonferroni multiple-comparison tests reinforce no differences between all pairs of categories of smoking dependence.

Next, we present the results from multivariable models (Table 4) that focused on the entire sample. These analyses, which control for sociodemographic and health indicators, indicate that individuals who are current smokers have lower scores for social participation (β = − 2.6, 95% CI -5.6, 0.3; *p* = 0.081) and intimacy (β = − 3.8, 95% CI -8.2, 0.5; *p* = 0.083). In general, men reported higher levels of QOL than women, particularly intimacy, social relationship/participation, psychological and environmental. Compared to oldest old (80 years of older), those who were younger reported higher QOL related to physical health, sensory abilities, autonomy, and past, present and future activities. However, those 60–69 years-old reported lower levels related to social relationship. Lower education and having more chronic conditions were associated with lower levels of QOL.

In the next set analyses (Table 5) the analyses are restricted to current smokers. Results indicate that smokers with medium levels of nicotine dependence had higher scores on social relationship than those with lower levels of dependence. On the other hand, smokers with high or very high dependence had lower scores for death and dying than those with low or very low dependence (β = − 10.6, 95% CI -19.9, − 1.4, *p* = 0.025), which indicate that those with higher levels of dependence are more concerned and afraid about death and pain before death. Longer exposure to smoking in years was associated with worse physical health. Among smokers, men also report higher levels of QOL than women. Smokers with lower educational levels reporting lower QOL related to environmental and death and dying than those with more education, but reported higher levels related to psychological and social relationship. Having five or more conditions are associated with worse levels of QOL among smokers.

In the last set of analyses (Table 6), we focus on past smokers and examine whether longer cessation time is associated with QOL. Results indicate that longer cessation time is positively associated (β = 0.2, 95% CI 0.0, 0.3, *p* = 0.007) with higher scores for the psychological health domain. Among those who had stopped smoking, men reported higher levels of QOL than women. Higher educational levels were protective within this group of past smokers, with those with higher education reporting higher levels of QOL. Having 5 or more health conditions was detrimental to one’s health among those who had quitted smoking.

## Discussion

This study examined the association between smoking, nicotine dependence, and duration of smoking cessation on QOL among older adults in an urban area in Brazil. In general, results pointed to a few differences between current smokers, past smokers, and never smokers. Nonetheless, current smokers had lower scores in the social participation and intimacy domains than those who never smoked. Among older adults who are current smokers, those with higher levels of nicotine dependence are more concerned with death and more afraid of having pain than smokers with low dependence levels. Evidence points to the positive impact of duration of cessation on psychological health. However, one unexpected result was related to higher levels of social relationship among those with medium dependence.

Previous studies in Brazil that have focused on adult smokers younger than 60 have found worse QOL among adult smokers, particularly in social aspects [29–31]. This finding is similar to this study, which also identified current smokers reporting worse social participation and intimacy scores. Previous studies have also pointed out to that higher levels of nicotine dependence are associated with lower quality of life, higher levels of disability and lower life expectancy [29, 32–34]. This study corroborates with this finding. This study showed that older adults who reported high or very high levels were associated with lower scores for the death and dying facet of WHOQOL-OLD, which is related to worries, concerns, and fears about death and dying [24]. Given the well-known connection between smoking and mortality, smokers tend to report more concerns about their health [35], which is intricately linked to fears about death. Smokers, particularly those with higher levels of nicotine dependence, seem to be even more exposed to those fears.

Our findings indicate that smokers with medium levels of nicotine dependence had higher scores for social relationship than those with lower levels. This is an unexpected finding given that older adults with more elevated levels of nicotine dependence are often more exposed to morbidity and disability, which could limit their social relationships [36]. However, it is important to note that individuals may use tobacco products to manage stress, mood and social acceptance [37]. Given the complex associations between smoking dependence, psychological factors and social engagement, further research is needed to identify the context in which these individuals sustain smoking and remain with higher levels of social engagement quality than those with lower nicotine dependence [38]. Given the negative impact of nicotine dependence on QOL, further studies are needed to estimate the prevalence of nicotine dependence in the Brazilian population and to examine the characteristics of those more vulnerable in developing it.

The average duration of smoking cessation was about 24 years and longer smoking cessation was positively associated with better psychological health. This confirms previous studies that have shown the positive effects of smoking cessation on QOL and on life expectancy [33, 39, 40]. However, the duration since quitting smoking was not associated with other domains and facets of QOL as shown in other studies [41]. Given the positive impact of smoking cessation on psychological health, training of health care professionals that can deliver messages related to the benefits of smoking cessation can be critical for reducing smoking rates. In addition, there is indication that Brazilian campaigns aimed at showing the negative health impact of continuing smoking have reached not only older adults, but also encouraged smoking cessation in younger generations [6]. Therefore, providing funds for these campaigns should be a priority as they have the potential to benefit younger cohorts and have a longer lasting effect.

Older men in our sample were more likely to have been exposed to smoking than women given that the tobacco epidemic started with men [6]. Men not only have higher prevalence of smoking, they are also more likely to have smoked in the past, which has been reported in other studies in Brazil [42]. In recent years, prevalence rates have decreased for both men and women [6]. Nonetheless, the absolute prevalence decreased more among men than women [6]. Therefore, efforts should target both groups as women became more exposed to smoking as the tobacco epidemic evolved. Even though men have historically being more exposed to smoking than women, older men in Brazil report higher levels of QOL. This finding corroborates with previous studies in Brazil that have found similar gender differences among adults and older adults [43]. Consistent with the findings from the general population, higher levels of QOL among men were also found among current and past smokers.

For the most part, older adults with higher levels of education reported better QOL. This finding confirms previous studies in Brazil that have shown that higher socioeconomic status, such as having enough money, are associated with better QOL among older adults [44]. Higher socioeconomic status if often associated with better living conditions and lower exposure to detrimental environments, better access to health care, as well as better quality of health care, which often lead to better health outcomes and quality of life [45, 46]. However, it is also possible that poor health in early life impacted their educational achievement. Therefore, there are many mechanisms that link health, quality of life and socioeconomic resources. Policies aimed at improving access to education and health at younger ages have the potential to improve QOL at older ages.

Results also show the strong effects of having more health conditions when reducing QOL. These findings corroborate with previous studies which show that cigarette smoking and having more chronic conditions are associated with health declines and a lower QOL among older people [47, 48]. Most differences in perceived health among smokers, past smokers, and never smokers were small, as previous studies in Brazil have also shown [35]. Prior research shows associations between QOL and health conditions for both smokers and never smokers [49]. This study finds that older adults with more health diseases have worse QOL. Smokers in the current study were exposed on average to about 50 years of smoking, which prior research associates with greater dependence on nicotine and consequently poorer physical and mental health [50]. Given the association between smoking and the development of chronic conditions, promotion of good health behaviors are important at reducing disease prevalence. At the same time, they also point to the need of improving health care as a way to better manage these conditions and their complications that can impair quality of life.

The study presents some limitations. First, the study is cross-sectional and we cannot address causality. Second, the data is limited to the urban area of one city in Brazil, which limits the generalizability of findings, particularly as it excludes rural residents who may differ from those in urban areas. Third, some variables such as social support and disabilities, previously shown to be associated with QOL, are not available in the dataset. Fourth, in this study we do not distinguish between cigarette smoking, cigar or pipe smoking. It is possible that differences across users may influence quality of life and may be associated with socioeconomic characteristics, such as gender and education. Another limitation is the adopted cutoff points for the MMSE, which vary across studies [51]. We adopted the cutoffs adjusted for schooling developed by Bertolucci and colleagues [22]. However, it is important to point out that, for most of the sample, the adopted cutoff was 18, which is expected to minimize the number of false-positives among the less educated. Bertolucci and colleagues found that specificity levels were above 96% and sensitivity above 76% at all educational levels. Other studies in Brazil with older adults also pointed out for the cutoff of 18 among those with lower education [52] and cutoff of 13 among older adults (> 65 years old) [53]. However, these cutoff points are lower than those adopted in more developed countries, so it is possible that some participants in our study have some mild to moderate cognitive limitations. Finally, similarly to the Brazilian population, the sample is composed mostly by women. This higher proportion of women at older ages reflect their higher survivorship, which can be influenced by their past smoking habits.

Previous studies in Brazil have shown that smoking can be detrimental to health and quality of life. Using a large sample and two measures of QOL, this study contributes to this literature by assessing how smoking, nicotine dependence, and duration of smoking cessation are associated with QOL among older adults in Brazil. Results from this study point to the importance of policies aimed at promoting smoking cessation, reducing smoking initiation and providing adequate treatment for older adults.

## Conclusion

This study highlights the negative impact of smoking and smoking exposure on QOL of older adults. Smoking cessation and treatment programs should be expanded and better target older adults in order to promote better QOL. In addition, campaigns targeting older adults should clearly point to the negative impact of tobacco use and the benefits of smoking cessation.

## Abbreviations

MMSE: Mini-Mental State Questionnaire
QOL: Quality of Life
WHOQOL-BREF: World Health Organization Brief Quality of Life
WHOQOL-OLD: World Health Organization Quality of Life Assessment for Older Adults
